# Cardiospecific CD36 suppression by lentivirus-mediated RNA interference prevents cardiac hypertrophy and systolic dysfunction in high-fat-diet induced obese mice

**DOI:** 10.1186/s12933-015-0234-z

**Published:** 2015-06-03

**Authors:** Yijie Zhang, Mingwei Bao, Mingyan Dai, Xin Wang, Wenbo He, Tuantuan Tan, Dandan Lin, Wei Wang, Ying Wen, Rui Zhang

**Affiliations:** Department of Cardiology, Wuhan University, Renmin Hospital, 238 Jiefang Road, Wuhan, 430060 Peoples Republic of China; Cardiovascular Research Institute of Wuhan University, 238 Jiefang Road, Wuhan, 430060 Peoples Republic of China; Central Laboratory of Renmin Hospital, Wuhan University, 238 Jiefang Road, Wuhan, 430060 Peoples Republic of China; Department of Ultrasonography, Wuhan University, Renmin Hospital, 238 Jiefang Road, Wuhan, 430060 Peoples Republic of China; Department of Oncology, Wuhan University, Renmin Hospital, 238 Jiefang Road, Wuhan, 430060 Peoples Republic of China; Department of Thoracic Surgery, Wuhan University, Renmin Hospital, 238 Jiefang Road, Wuhan, 430060 Peoples Republic of China

**Keywords:** Obesity, Cardiomyopathy, CD36, RNA interference, Lipotoxicity

## Abstract

**Background:**

Fatty acid (FA) catabolism abnormality has been proved to play an important role in obesity-related cardiomyopathy. We hypothesized that cardiospecific suppression of CD36, the predominant membrane FA transporter, would protect against obesity-related cardiomyopathy.

**Methods:**

Four-wk-old male C57BL/6 J mice were fed with either high-fat-diet (HFD) or control-normal-diet for 2 wk. Then they were subjected to intramyocardial injection with recombinant lentiviral vectors containing short hairpin RNAs to selectively downregulate the expression of either cardiac CD36 or irrelevant gene by RNA interference. After a 10-wk continuation of the diet, biochemical, functional, morphological, histological, metabolic and molecular profiles were assessed.

**Results:**

HFD administration elicited obesity, cardiac hypertrophy and systolic dysfunction accompanied with elevated serum levels of blood urea nitrogen (BUN), creatinine, fasting serum glucose (FSG), total cholesterol (TC) and triglyceride. Additionally, HFD consumption promoted lipid accumulation and reactive oxygen species (ROS) generation in the cardiomyocytes. Cardiospecific CD36 inhibition protected against HFD induced cardiac remodeling by decreasing heart/body weight ratio, increasing left ventricular (LV) ejection fraction and fractional shortening as well as normalizing LV diameter, without influencing body weight gain. Inhibition of cardiac CD36 also mitigated obesity induced alteration in BUN, creatinine and triglyceride, but had no effect on FSG or TC. Moreover, cardiospecific CD36 deficiency corrected myocardial lipid overaccumulation and intracellular ROS overproduction that were induced by HFD feeding.

**Conclusions:**

Cardiospecific CD36 inhibition protects against the aggravation of cardiac functional and morphological changes associated with HFD induced obesity. CD36 represents a potential therapeutic target for obesity cardiomyopathy.

## Background

With the spread of sedentary lifestyle, obesity becomes a major global health issue. It is regarded as an energy balance disorder in which inappropriate expansion and dysfunction of adipose tissue leading to a pandemic of type 2 diabetes [1] and increased risk of developing cardiovascular diseases [2, 3]. Accumulated experimental evidence supports an association between obesity and morphological and functional changes in hearts of both humans [3–6] and animals [7–9]. Many of these changes, such as cardiac hypertrophy, compromised left ventricular (LV) function, are believed to be precursors of more overt forms of cardiac dysfunction and heart failure. Most investigations evaluating the relation of obesity to cardiac remodeling have focused on the role of hemodynamics and cardiovascular comorbidities. For the past few years, certain metabolic disorders, observed mainly in animal models, have attracted increasing attention [9–14].

Cardiomyocytes are metabolically flexible. They predominantly use fatty acid (FA), but can also use glucose, lactate and any other available substrates to maintain a steady level of ATP required for contraction [15]. This flexibility in substrate use is important for normal cardiac function and its alteration by obesity or diabetes will contribute to cardiomyopathy [16].

CD36 is a pivotal FA transporter which is responsible for the majority of FA uptake in both rodent [17–19] and human hearts [20–22]. Under physiological conditions, CD36 is distributed equally between intracellular storage compartments and the sarcolemma. However, obesity has been proved to cause a permanent translocation of CD36 from intracellular storage compartments to the sarcolemma, leading to the excessive FA uptake into the heart [23, 24]. Once FA has entered the cardiomyocytes, it can be used for ATP production by mitochondrial β-oxidation or be stored as natural lipids (mostly triglycerides). Thereby, in response to increased myocardial FA intake, FA becomes almost the only substrate for cardiomyocytes which may ultimately lead to cardiac metabolic inflexibility, lipotoxicity [25], oxidative stress [26] and subsequent development of cardiomyopathy [16].

Based on the key role of CD36 in the initiation of obesity-related cardiomyopathy, we hypothesized that cardiospecific CD36 deficiency in the setting of obesity would attenuate cardiac remodeling and dysfunction by reducing lipotoxicity and oxidative stress. To test this hypothesis, we employed short hairpin RNAs (shRNAs) to selectively downregulate the expression of CD36 in the heart, and decrease the FA uptake process, then observe whether this intervention was sufficient to prevent myocardial lipotoxicity and cardiac dysfunction. Obesity was resulted from exposure to a high-fat-diet (HFD) for 12 wk in this study.

## Materials and Methods

### Animals

Age matched male C57BL/6 J mice were purchased from Beijing HFK Bioscience Co., Ltd (Beijing, China). Animals were housed 4 per cage at constant temperature (22 ± 2 °C) and humidity (45-50 %), with a 12-hr light/dark cycle and unrestricted access to food and water. If required, animals were lightly anesthetized with 2 % isoflurane. When underwent intramyocardial injection or sacrificed, animals were deeply anesthetized with 1 % pentobarbital sodium (9 μg/g, i.p.). All procedures were performed in accordance with the “Guide for the Care and Use of Laboratory Animals” published by the US National Institutes of Health (NIH Publication no. 85–23, revised 1996) and approved by the Institutional Animal Care and Use Committee at Renmin Hospital, Wuhan University, China.

Mice were randomized into 4 groups; normal mice with cardiac CD36 (N-CD36) or irrelevant gene (N-mock) silencing, obese mice with cardiac CD36 (O-CD36) or irrelevant gene (O-mock) silencing. Green fluorescent protein (GFP) gene served as irrelevant gene in this study. Animals were fed a control-normal-diet (CND) post weaning until 4 wk of age. Then they were either continued on CND (normal mice) or switched to HFD (obese mice) for a further 12 wk. CND contained 10 % fat, 20 % protein, and 70 % carbohydrate (D12450B, Beijing HFK Bioscience, China), while HFD was consisted of 60 % fat, 20 % protein, and 20 % carbohydrate (D12492, Beijing HFK Bioscience). The gross energy for CND and HFD was 16.10 and 21.95 kJ/g, respectively. At 6 wk of age, mice were subjected to intramyocardial injection with recombinant lentiviral vectors containing shRNAs to downregulate the expression of either cardiac CD36 or GFP. Body weight was measured weekly. Cardiac function, morphology, biochemical parameters, myocardial lipid content and reactive oxygen species (ROS) profiles were assessed when they reached 16 wk of age.

### Preparation of lentivirus

Lentivirus used in this study was kindly provided by Dr. Liang Chu from State Key Laboratory of Cell Biology, Institute of Biochemistry and Cell Biology, Shanghai Institutes for Biological Sciences, Chinese Academy of Sciences (Shanghai, China). ShRNAs targeting murine CD36 (5’-CCG GCA GTC GGA GAC ATG CTT ATC TCG AGA TAA GCA TGT CTC CGA CTG TTT TTG-3’) were synthesized and cloned into pLKO.1-puro (SHC001, Sigma-Aldrich, USA) to generate the lentiviral expression vectors, which were then transfected into 293 T cells with packaging plasmids pCMV-VSV-G (#8454, Addgene plasmid) and pCMV-dR8.2 (#8455, Addgene plasmid). Viral supernatant was harvested 48 h after transfection and the titer was detected. ShRNAs targeting GFP (5’-CCG GTC ACC TTC ACC CTC TCC ACT TCT CGA GAA GTG GAG AGG GTG AAG GTG ATT TTT G -3’) served as negative control.

### Intramyocardial injection

To deliver the lentiviral vectors into myocardium, we employed a rapid surgical method according to the protocol from Erhe Gao [27] with modification. Briefly, 6-wk-old mice were subjected to small left thoracotomy followed by temporary cardio-exteriorization. A 25 μL gastight microliter syringe equipped with a 31-gauge needle (Shanghai Gaoge, China) was used for the injection. The needle tip was inserted into apex cordis with an approximately 30° angle and advanced in parallel with the ventricular wall until reached 3 mm into the myocardium. A total of 10 μL lentivirus (1 × 10^7^ particles per milliliter) was injected. The heart was immediately placed back into the thoracic cavity with thoracic air evacuation to avoid pneumothorax.

### Echocardiography

Transthoracic echocardiograms were performed on anesthetized mice using a MyLab™ 30 CV ultrasound (Biosound Esaote, Italy) equipped with a 10 MHz transducer. Parameters were obtained and recorded in a digital format. Images were analyzed off-line by a researcher blinded to the grouping. LV ejection fraction (LVEF) and fractional shortening (LVFS), which quantify muscle function and contractibility of the ventricular wall, were calculated.

### Electrocardiography

Mice were lightly anesthetized followed by the connection of electrocardiographic electrodes to the limbs. Parameters were obtained and recorded with a MP150 electrocardiograph (Biopac, USA). Acqknowledge 4.1 version software was used for the parameters measurement by a researcher blinded to the grouping.

### Quantitative Real-Time RT-PCR

Total RNA was isolated from heart tissue using the TRIzol isolation method according to the manufacturer’s protocol (15996–026, Invirtrogen, USA). First strand cDNA synthesis was performed according to the protocol of the ReverTra AceR qPCR RT kit (FSQ-101, Toyobo, China). Quantitative real-time RT-PCR, using SYBR Green detection chemistry (FP205, Tiagen, China), was performed on a CFX96 Touch™ Real-Time PCR Detection System (Bio-Rad, USA). All reactions were done in triplicate. GAPDH was used as the invariant control. The sequences of the qRT-PCR primers are as follows: CD36 forward primer (5’-AGC AAC TGG TGG ATG GTT TC-3’) and reverse primer (5’-TCA AGG GAG AGC ACT GGT TT-3’); GAPDH forward primer (5’-CTC ATG ACC ACA GTC CAT GC-3’) and reverse primer (5’-GGA TGA CCT TGC CCA CAG CC-3’).

### Immunoblotting

LV homogenates were lysed in RIPA lysis buffer (sc-24948, Santa Cruz, USA). The lysates were used for protein detection by SDS-polyacrylamide gel electrophoresis, followed by western blotting. The primary antibodies used in this study included CD36 (sc-9154, Santa Cruz, 1:200 dilution) and GAPDH (CW0100A, Cwbiotech, China, 1:1 000 dilution).

### Serum Analysis

Blood samples were obtained by cardiac puncture from mice with an overnight fasting. Serum biochemical parameters were analyzed at clinical laboratory of Renmin Hospital of Wuhan University, using a Advia 2400 automatic biochemical analyzer (Siemens, Germany) and kits for alanine aminotransferase (ALT), aspartate transaminase (AST), alkaline phosphatase (ALP), albumin, blood urea nitrogen (BUN), creatinine, fasting serum glucose (FSG), total cholesterol (TC) and triglyceride.

### Cardiac neutral lipid content

Excess FA in the heart is stored in droplets as neutral lipid, which can be assessed by oil red O staining. Frozen LV sections (7 μm) were stained with oil red O and counterstained with hematoxylin. Images were captured at × 400 by Eclipse Ci-E microscope (Nikon, Japan). Image-Pro Plus 6.0 version software was used for the analysis of lipid content.

### Isolation of cardiomyocytes

Cardiomyocytes were isolated by enzymatic dissociation method [28]. Fresh heart was rapidly removed and perfused with oxygenated Ca^2+^-free Tyrode’s solution (135 mmol/L NaCl, 5.4 mmol/L KCl, 0.33 mmol/L NaH_2_PO_4_, 10 mmol/L HEPES, 10 mmol/L Glucose, and 1 mmol/L MgCl_2_, pH = 7.35 at 37 °C) and then with added collagenase type II (C6885-1G, Sigma-Aldrich, 0.33 mg/ml). After perfusion, the ventricle was minced, gently agitated, filtered with 150 μm nylon mesh and maintained at room temperature.

### Measurement of ROS

Dihydroethidium (DHE, D7008, Sigma-Aldrich) was utilized to evaluate *in situ* production of ROS. Unfixed cryosections (10 μm) were stained with DHE (10 μmol/L) at 37 °C for 30 min in a humidified dark chamber. Ethidium fluorescence (excitation/emission at 488/610 nm) was examined by fluorescence microscopy (Eclipse Ti-SR, Nikon) at × 200 and evaluated by using Image-Pro Plus 6.0 version.

To assess the production of ROS by a second approach, isolated cardiomyocytes were prepared as described above and incubated with 2’,7’-dichlorofluorescein diacetate (DCFH-DA, S0033, Beyotime, China, 10 μmol/L) at 37 °C for 20 min. Mean fluorescence intensity (excitation/emission at 488/525 nm) which mirrored the levels of intracellular ROS was analyzed by flow cytometry (FACSCalibur, BD, USA).

### Statistics

Numerical values were presented as means ± SD. Student’s *t*-test was used to compare mean values between normal and obese mice. Multiple group comparisons were evaluated via one-way ANOVA followed by a Student-Newman-Keuls post hoc test. SPSS 17.0 version software was used for the statistical analysis. Statistical significance was defined as *P* < 0.05 and tests were performed two sides.

## Results

### HFD feeding induced obesity and altered biochemical parameters

**Fig. 1:**
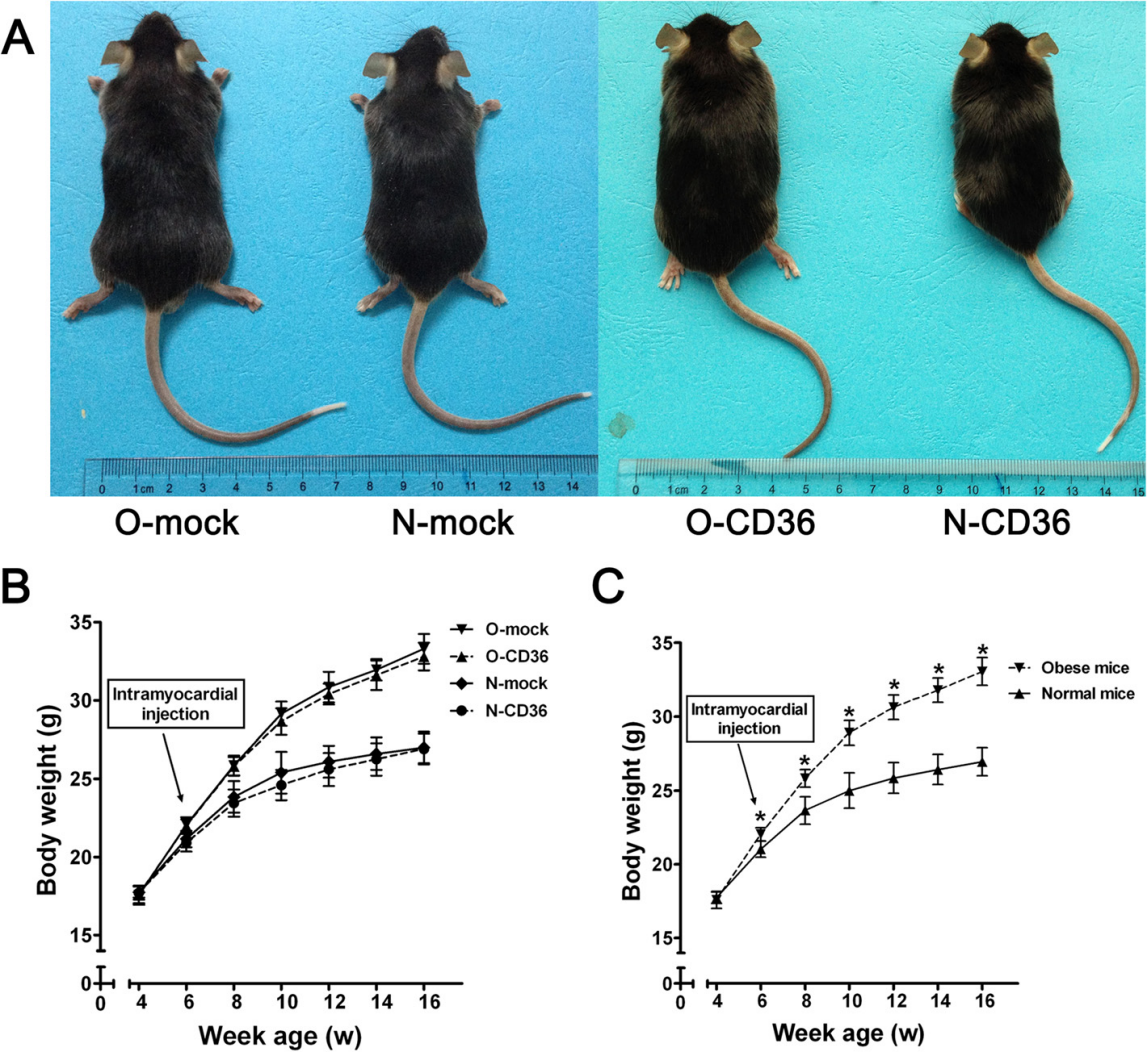
Phenotype and weight gain. **a** Phenotype of 16-wk-old mice after 12 wk of diet administration. O-mock and O-CD36, Typical 16-wk-old C57BL/6 J mice with HFD feeding. N-mock and N-CD36, 16-wk-old siblings after 12 wk of CND. **b** and **c**, Effects of different feeding protocols on weight gain. Mice in O-mock and O-CD36 groups were merged as obese mice, while in N-mock and N-CD36 groups were merged as normal mice in **c**. Values are mean ± SD, *N* = 10 for each of the four groups. ^*^ *P* < 0.001 vs. normal mice

After a 12-wk HFD feeding, mice exhibited obvious obesity that was characterized by significant increase in body weight (33.05 ± 0.93 g for obese mice compared to 26.95 ± 0.96 g for normal mice, *P* < 0.001). Cardiospecific CD36 silencing did not affect body weight regardless of the diet regimen (Fig. 1). To more robustly characterize the effect of HFD feeding and cardiac CD36 suppression on phenotype, representative serum biochemical parameters were examined. We found mice receiving HFD had higher levels of BUN, creatinine, FSG, triglyceride and TC than mice receiving CND, whereas the levels of ALT, AST and ALP remained comparable among all experimental groups. Interestingly, cardiac CD36 deficiency decreased the levels of BUN and creatinine in HFD fed mice. Besides, the inhibition of CD36 also lowered serum triglyceride level of obese mice to normal level, without influencing FSG or TC concentration. These data and additional parameters are summarized in Table 1.

**Table 1:** Biometric and biochemical parameters

Phenotypic variables	N-mock	N-CD36	O-mock	O-CD36
Body weight (g)	27.00 ± 1.00	26.90 ± 0.97	33.30 ± 0.95^*a^	32.80 ± 0.89^*a^
Alanine aminotransferase (U/L)	31.00 ± 2.93	33.25 ± 3.73	33.25 ± 3.96	34.50 ± 4.63
Aspartate transaminase (U/L)	112.00 ± 20.67	116.75 ± 19.02	118.38 ± 12.83	118.13 ± 14.75
Alkaline phosphatase (U/L)	86.25 ± 17.39	92.50 ± 8.77	104.25 ± 11.85	100.75 ± 14.75
Albumin (g/L)	27.75 ± 1.06	28.70 ± 1.04	25.70 ± 1.55^*a^	26.63 ± 1.54^a^
Blood urea nitrogen (mmol/L)	11.10 ± 0.34	12.51 ± 1.37	13.66 ± 2.25^*^	10.44 ± 2.31^b^
Creatinine (μmol/L)	10.75 ± 0.46	12.50 ± 1.77	14.38 ± 3.42^*^	10.13 ± 1.96^b^
Fasting serum glucose (mmol/L)	4.93 ± 0.29	4.76 ± 1.26	6.63 ± 1.84^*a^	6.42 ± 1.32^*a^
Total cholesterol (mmol/L)	2.01 ± 0.11	2.15 ± 0.13	4.74 ± 0.84^*a^	4.64 ± 0.79^*a^
Triglyceride (mmol/L)	0.92 ± 0.14	0.85 ± 0.09	2.38 ± 1.38^*a^	1.77 ± 0.66

Values are mean ± SD, *N* = 8-10 for each group

^a^ *P* < 0.05 vs. N-CD36

^b^ *P* < 0.05 vs. O-mock

^*^ *P* < 0.05 vs. N-mock

### Lentivirus-mediated RNAi efficiently downregulated the expression of CD36 in heart tissues

**Fig. 2:**
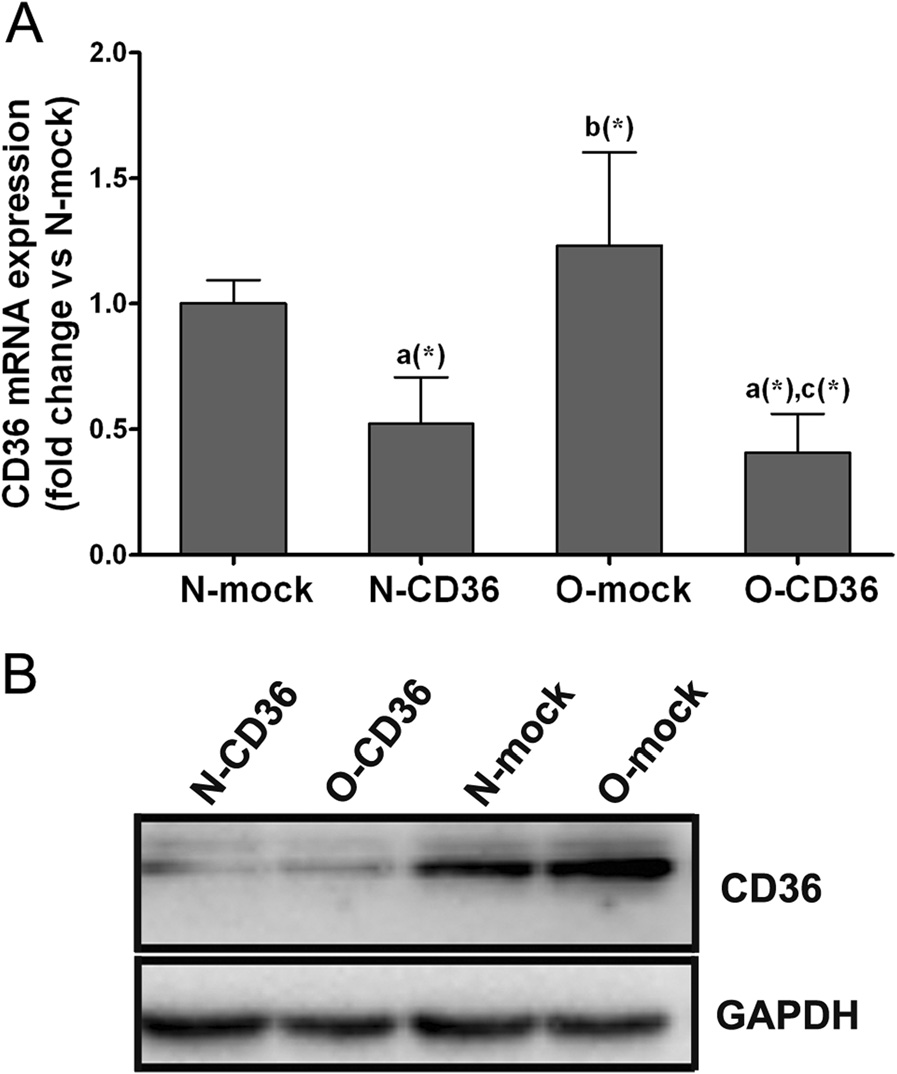
Effect of RNAi in the setting of HFD feeding on CD36 expression. **a** Quantitative real-time RT-PCR for cardiac CD36 mRNA expression. Lentivirus mediated CD36 RNAi using short hairpin RNAs (shRNAs) lead to the downregulation of CD36. Mice in groups treated with sh-CD36 (N-CD36 and O-CD36) showed decreased CD36 mRNA expression by nearly 50 % in comparison with groups treated with sh-GFP (N-mock and O-mock). **b** Western blot analysis of heart tissue lysates from each group. GAPDH was introduced as an internal reference. Values are mean ± SD, *N* = 3 for each group. *a*, vs. N-mock; *b*, vs. N-CD36; *c*, vs. O-mock; (*) *P* < 0.05

Quantitative real-time RT-PCR revealed that the level of CD36 mRNA in mice hearts was not significantly altered by HFD induced obesity (Fig. 2a). By utilizing RNAi technique, the cardiac CD36 mRNA was markedly lowered to 40 % - 50 % of control levels in both CND and HFD fed mice (Fig. 2a). These data were well consistent with the immunoblotting patterns demonstrating lower CD36 protein levels in N-CD36/O-CD36 mice hearts compared with that of N-mock/O-mock (Fig. 2b). Our data illustrated that RNAi effectively downregulated the expression of CD36 in heart tissues.

### Cardiospecific CD36 silencing normalized heart hypertrophy in obese mice

**Fig. 3:**
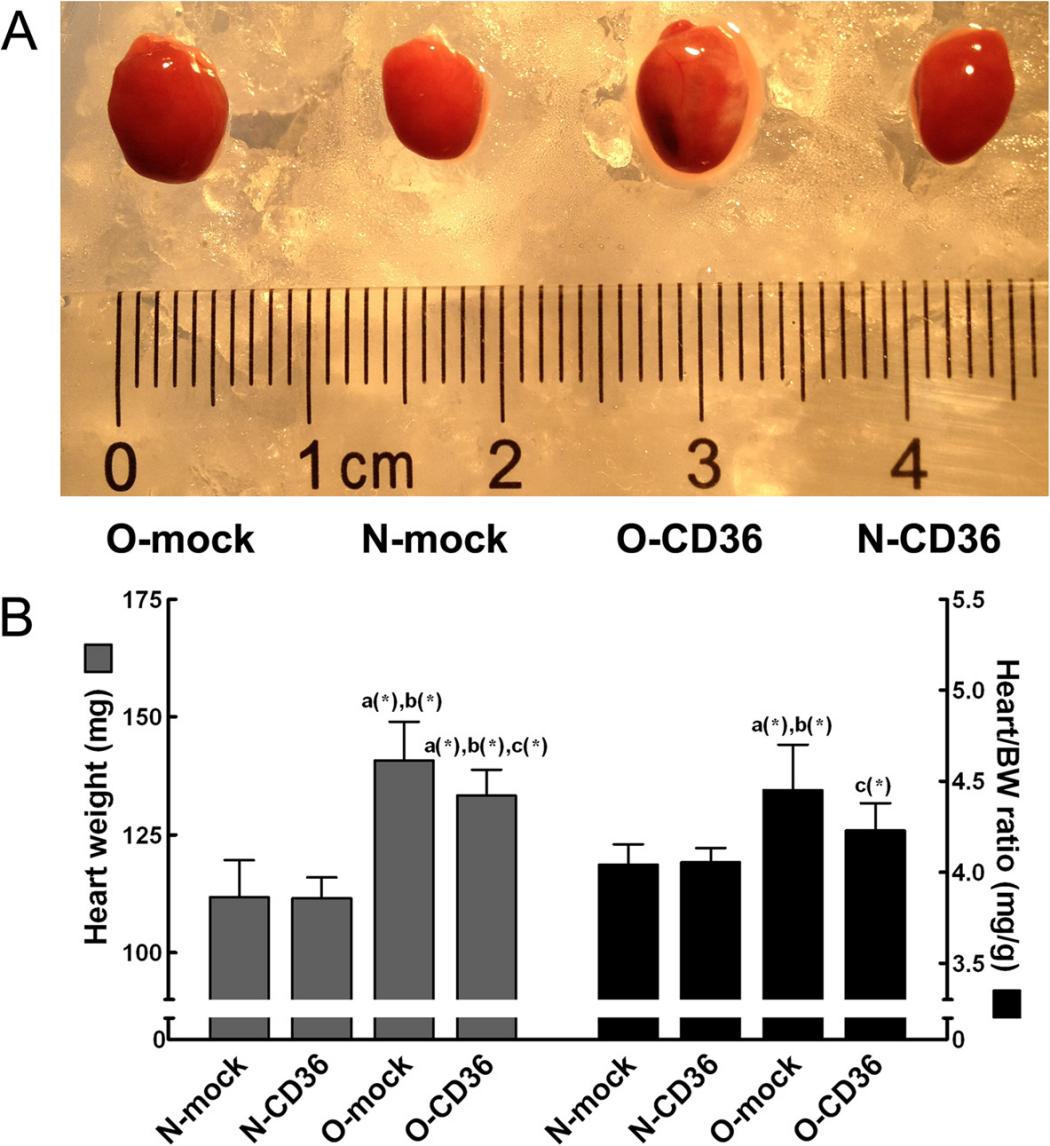
Phenotype of heart. **a** Gross appearance of a representative heart from each group. HFD induced visually cardiac hypertrophy compared to CND. Excised hearts were arrested in diastole with 10 % KCl. **b** Gross heart weight and heart/BW ratio revealed significant increase with HFD compared to CND. Cardiospecific CD36 inhibition significantly decreased heart/BW ratio. BW: body weight. Values are mean ± SD, *N* = 8 for each group. *a*, vs. N-mock; *b*, vs. N-CD36; *c*, vs. O-mock; (*) *P* < 0.05

Obesity cardiomyopathy is characterized by cardiac hypertrophy, a phenotype that is commonly observed in both obese humans and animals [4, 29]. To investigate the effects of CD36 deficiency on cardiac hypertrophy, the hearts were excised and weighted. The heart weight of 16-wk-old O-mock mice was significantly higher than that of N-mock mice. Cardiac CD36 suppression in O-CD36 mice significantly reduced heart weight but not back to normal level (Fig. 3). Heart/body weight ratio (heart/BW ratio) was calculated in this study to normalize weights and eliminate confounding effects of differences in size. As shown in Fig. 3b, HFD feeding caused a significant increase in heart/BW ratio in O-mock mice compared to N-mock and N-CD36 mice. Surprisingly, cardiac CD36 RNAi completely normalized heart/BW ratio back to normal level.

### Obesity-related systolic dysfunction was ameliorated by cardiac CD36 silencing

**Fig. 4:**
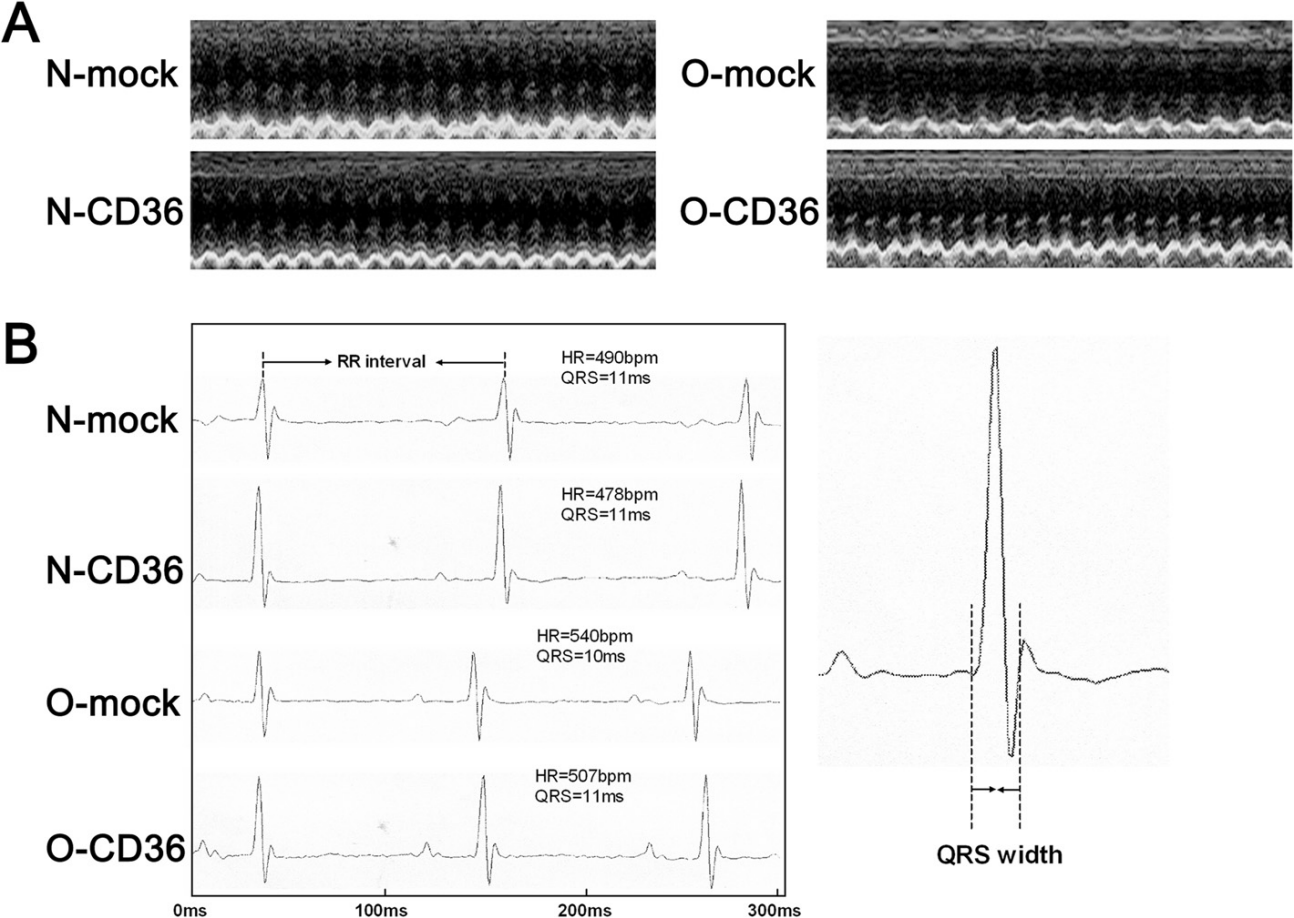
Echocardiogram and electrocardiogram. **a** Representative transthoracic echocardiograms for each of the four groups. **b** Representative electrocardiograms for each of the four groups and the method of QRS width measurement

After 12 wk of diet intervention, mice in O-mock group revealed mild but significant LV systolic dysfunction (depressed LVEF and LVFS on M-mode images). In striking contrast, LV systolic function of the O-CD36 mice was not different from that of the N-mock and N-CD36 mice (Table 2). These changes in LVEF and LVFS were reflected mainly in increased LV diameter. Obesity modestly but significantly enlarged LV end-diastolic diameter (LVEDd) but was corrected by CD36 deficiency. Additionally, LV end-systolic diameter (LVESd) was significantly increased after a 12-wk HFD, CD36 deficiency showed a trend to blunt this alteration with no statistical significance. However, LV posterior wall thickness and QRS-complex width were not altered by HFD feeding and no effects were seen with cardiac CD36 inhibition (Table 2). Figure 4 exhibits representative echocardiograms and electrocardiograms for each of the four groups. Taken together with the cardiac hypertrophic growth data, these results indicate that the inhibition of cardiac CD36 protects against obesity-related cardiac hypertrophy and systolic dysfunction.

**Table 2:** Echocardiographic and electrocardiographic parameters

Phenotypic variables	N-mock	N-CD36	O-mock	O-CD36
Heart rate (bpm)	488.75 ± 31.01	495.25 ± 14.35	443.75 ± 52.31	446.25 ± 36.69
LVEDd (mm)	3.89 ± 0.12	3.91 ± 0.18	4.07 ± 0.12^*a^	3.86 ± 0.09^b^
LVESd (mm)	2.43 ± 0.19	2.40 ± 0.22	2.63 ± 0.12^*a^	2.48 ± 0.07
LVPWd (mm)	0.67 ± 0.03	0.67 ± 0.06	0.70 ± 0.04	0.68 ± 0.02
LVPWs (mm)	1.08 ± 0.05	1.08 ± 0.05	1.13 ± 0.05	1.13 ± 0.05
Ejection fraction (%)	74.75 ± 3.54	75.38 ± 1.60	70.25 ± 1.58^*a^	72.88 ± 2.36^b^
Fractional shortening (%)	37.75 ± 2.76	38.00 ± 1.07	34.25 ± 0.89^*a^	36.38 ± 1.30^b^
QRS-complex width (ms)	10.30 ± 0.67	9.60 ± 0.70	10.17 ± 0.72	9.75 ± 0.62

Values are mean ± SD, *N* = 8-12 for each group

*LVEDd* left ventricular end-diastolic diameter, *LVESd* left ventricular end-systolic diameter, *LVPWd* left ventricular posterior wall thickness at end-diastole, *LVPWs* left ventricular posterior wall thickness at end-systole

^a^ *P* < 0.05 vs. N-CD36

^b^ *P* < 0.05 vs. O-mock

^*^ *P* < 0.05 vs. N-mock

### Cardiospecific CD36 silencing abolished excessive myocardial lipid accumulation associated with obesity

**Fig. 5:**
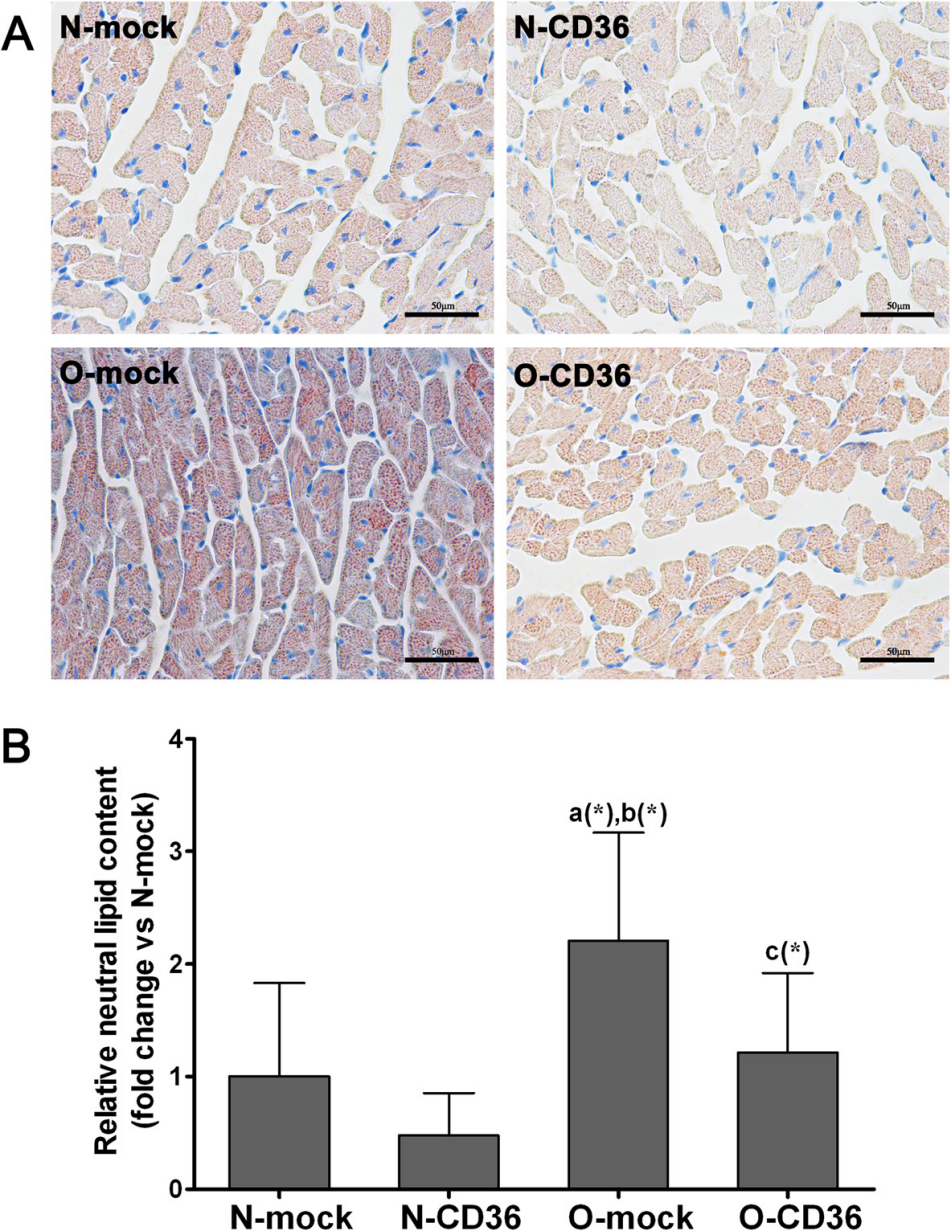
HFD feeding resulted in excessive myocardial lipid accumulation that was prevented by CD36 inhibition. **a** Representative images of myocardial staining for oil red O, a marker of neutral lipid. Red droplets indicate neutral lipid staining (scale bar indicates 50 μm). **b** Quantification of natural lipid revealed significant increase in lipid accumulation in mice hearts from O-mock compared to all other groups. Inhibition of cardiac CD36 abrogated the increase in lipid accumulation. Values are mean ± SD, three pictures of each mouse were analyzed and *N* = 3–5 for each group. *a*, vs. N-mock; *b*, vs. N-CD36; *c*, vs. O-mock; (*) *P* < 0.05

Increased lipogenesis is largely associated with lipotoxicity of obesity [30]. To determine the role of CD36 in myocardial lipid accumulation that occurs in obese mice, the effects of a 12-wk HFD feeding were analyzed. Oil red O staining demonstrated higher neutral lipid content (2.2 fold) in the mice hearts from O-mock group compared with N-mock group (Fig. 5). This confirms that HFD promotes intramyocardial lipid accumulation. In stark contrast, cardiac CD36 suppression significantly lowered oil red O staining intensity in O-CD36 mice hearts and back to normal level (Fig. 5).

### Cardiospecific CD36 silencing attenuated ROS generation in cardiac tissues and isolated cardiomyocytes from mice model of obesity

**Fig. 6:**
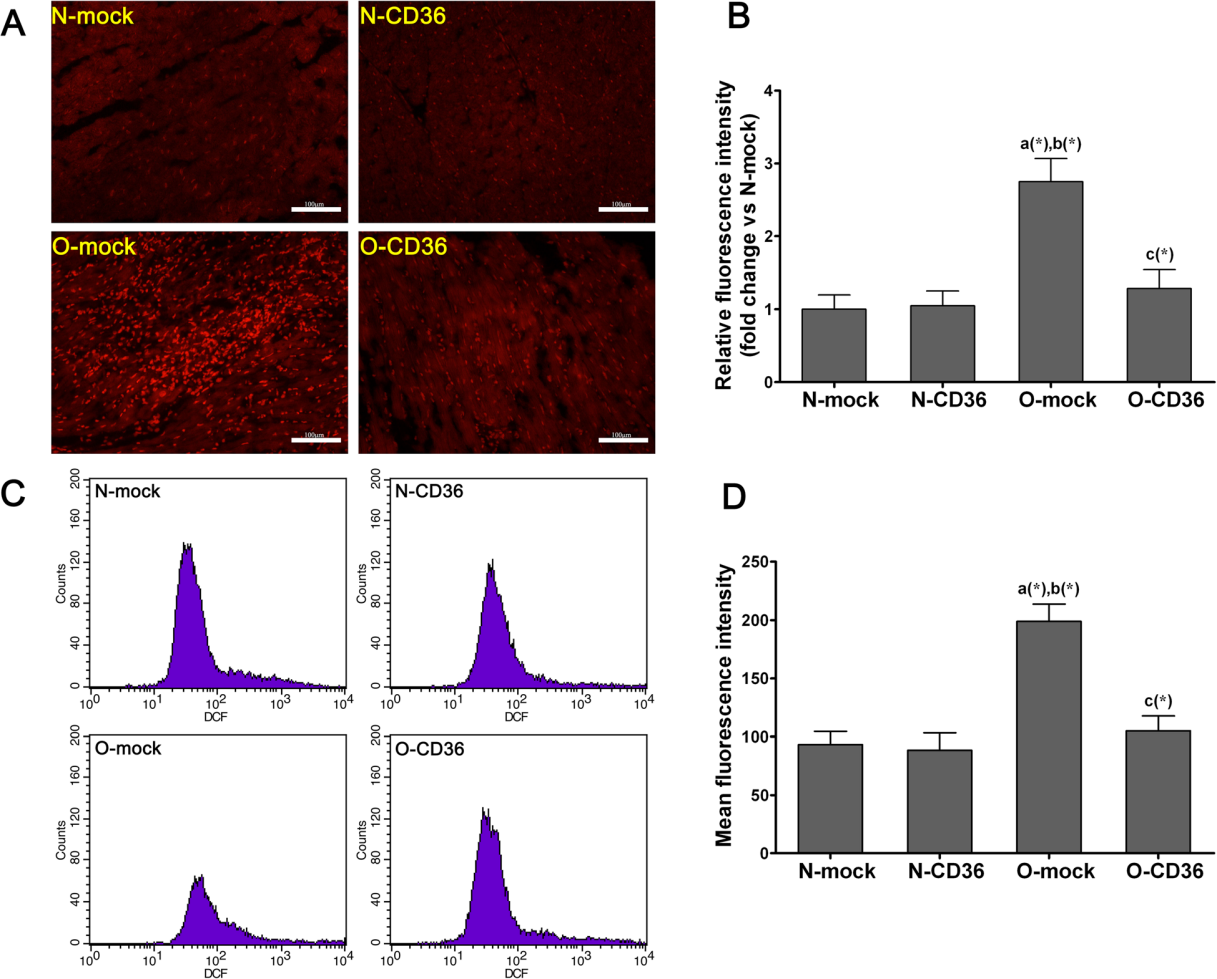
Cardiospecific CD36 silencing attenuated abnormal cardiac ROS generation in HFD fed mice. **a** and **b** Determination of in situ ROS generation in cardiac tissue by DHE staining. **a** Representative fluorescence microscopic images of DHE staining (red color) of cardiac frozen sections from each of the four groups (scale bar indicates 100 μm). **b** Relative fluorescence intensity which stands for levels of ROS generation. **c** and **d** Evaluation of ROS generation in isolated ventricular myocytes by DCFH-DA staining. **c** Isolated ventricular myocytes were incubated with DCFH-DA, and analyzed by flow cytometry. **d** Mean fluorescence intensity which stands for levels of ROS production. Quantitative and statistical analyses revealed significant increase in cardiac ROS productions in obese mice, but this was reconciled by selectively CD36 silencing. Values are mean ± SD, three pictures of each mouse were analyzed and *N* = 3 for each group in the DHE staining. A total of 100 000 events were analyzed for each mouse and *N* = 4 for each group in the DCFH-DA staining. *a*, vs. N-mock; *b*, vs. N-CD36; *c*, vs. O-mock; (*) *P* < 0.05

As demonstrated in Fig. 6a and b, ROS generation in frozen cardiac tissue sections was examined. DHE fluorescence staining intensity which is a specific indicator of ROS was found dramatically increased in O-mock mice (2.7 fold). Cardiospecific CD36 inhibition significantly decreased ROS concentration in O-CD36 mice and back to normal level. To reaffirm the intracellular ROS production in another way, isolated cardiomyocytes were incubated with DCFH-DA and analyzed by flow cytometry. In agreement with DHE staining, intracellular ROS formation was augmented in isolated cardiomyocytes from O-mock mice (2.1 fold), but this was completely abrogated by cardiac CD36 silencing (Fig. 6c and d).

## Discussion

HFD induced obese model has been widely used in the past, and is characterized by increased weight gain, elevated serum levels of FA and triglyceride [8, 13, 29, 31]. Here we demonstrate a tight association between myocardial FA metabolic disorder and obesity-related cardiac remodeling. The adverse effect of HFD-induced obesity on cardiac function was prevented by inhibition of the FA transporter CD36. Furthermore, we revealed that myocardial lipid overaccumulation and intracellular ROS overproduction appears to be important denominators for obesity cardiomyopathy.

### Myocardial metabolic disorder in obese mice

In obesity and its related conditions, plasma FA and triglyceride concentrations are elevated. Meanwhile, the process of myocardial FA uptake is dramatically enhanced by the relocation of CD36 to the sarcolemma [23, 24]. In response, the rate of FA oxidation will increase, but eventually the rate of FA uptake will still exceed the rate of its oxidation. This mismatch between supply and utilization will lead to myocardial lipid deposition, and is further promoted by the exhaustion of oxidative capacity [18]. Longstanding lipid accumulation in the heart can ultimately culminate in the development of myocardial lipotoxicity [32]. Additionally, enhanced FA oxidation leads to an overproduction of ROS [14, 33, 34], a byproduct of FA peroxidation which plays an important role in increased oxidative stress as well as diminished energy for myocardial function [35]. Studies have repeatedly shown that lipotoxicity and oxidative stress are major causal factors for obesity cardiomyopathy [14, 18, 36, 37].

### Effect of cardiospecific CD36 inhibition on cardiac remodeling secondary to obesity

In this study, we demonstrated that chronic overnutrition with a HFD resulted in obesity and impaired biochemical parameters. We also detected a 27 % increase in cardiac mass (Fig. 3) accompanied with a 10 % decrease in systolic function (Fig. 4a and Table 2) in mice with HFD administration. These observations are consistent with previous findings from both human [3–6] and experimental obesity [7–9]. The deleterious effect of HFD-induced cardiac hypertrophy and systolic dysfunction was prevented by CD36 suppression. Thus, our results revealed a tight association between CD36 and cardiac remodeling in the setting of obesity. HFD consumption induced myocardial structural and functional changes were most probably associated with the increased FA uptake mediated by CD36.

CD36 is ubiquitously expressed in many tissues, such as adipose tissue, myocardium and skeletal muscle [19]. In order to simplify the complexities involved in systemic CD36 silencing, we employed directional intervention method to selectively downregulate the expression of cardiac CD36 in this investigation. As a result, targeted CD36 silencing did not worsen the biometric and biochemical parameters (Table 1), suggesting that our intervention *per se* did not adversely affecting the viability of the animals.

### Effects of CD36 on cardiac lipid deposition and intracellular ROS generation

Our intervention strategies aimed at limiting CD36-mediated myocardial FA uptake, which in turn would decelerate FA esterified into triglyceride and intracellular FA oxidation, thereby lowering intramyocardial lipid storage and excess production of ROS. Histological staining results suggest that mice with HFD consumption exhibited a 2.2 fold increased in cardiac lipid deposition. A similar degree of lipid content was reported in mice exposed to a similar diet for 16 wk [38]. Interestingly, cardiospecific CD36 deficiency corrected myocardial lipid overaccumulation elicited by HFD (Fig. 5). This finding was somewhat surprising given the existence of other cardiac FA transporters such as fatty acyl-CoA synthetase 1 and fatty acid transport protein 1. Our results provide additional evidence for the importance of CD36 as a cardiac FA transporter and the indispensable of CD36 in the development of obesity-related myocardial lipid accumulation.

In the process of normal mitochondrial respiration, a small amount of electrons are leaked to O_2_ at several sites of the electron transport chain, where ROS are generated [39]. Excessive FA accumulation and metabolism can trigger ROS formation, as a result of accelerated electrons flux through the β-oxidation [26]. St-Pierre and colleagues have demonstrated that increased oxidative stress correlates with lipid overload, suggesting a role for FA in the generation of ROS [34]. Indeed, mice with HFD consumption showed marked increase in ROS generation (2.7 fold and 2.1 fold for different detection methods, respectively), which was completely normalized by cardiac CD36 deficiency in this study (Fig. 6). The improvement in myocardial ROS generation in combination with amelioration in systolic function suggests that cardiospecific CD36 inhibition may improve cardiac function by decreasing delivery of oxidizing agents to the myocardium. Excessive generation and insufficient removal of ROS may promote oxidative damage to both mitochondria and mitochondria uncoupling that result in impaired ATP synthesis [40]. Thereby, reduced ATP synthesis may result in a cardiac energy deficit and contribute to systolic dysfunction [41]. Additionally, ROS have been implicated in the initiation of maladaptive events associated with apoptosis, calcium mishandling, endoplasmic reticulum stress, cardiac fibrosis, among others [14, 26, 42–44]. Hence, it is not surprising that prevention of ROS generation by cardiac CD36 inhibition has beneficial effects on cardiac function. The results shown here demonstrate that oxidative stress is a common feature linking obesity-induced FA metabolic disturbance to subsequent cardiomyopathy.

### Role of CD36 under different situations

CD36-mediated FA uptake is a fundamental life process. It’s not surprising that up- or down-regulated function of CD36 may play different roles in different tissues or under different conditions.

As reported by Tardiff K and colleagues, lentivirus-mediated RNAi directed against nestin significantly reduced protein expression and concomitantly attenuated basal DNA synthesis [45]. In our investigation, we did not observe the DNA synthesis of CD36 or other genes. To our knowledge, lentivirus can help to integrate exogenous shRNAs into host gene, leading to the downregulation of DNA synthesis. We did not measure the DNA synthesis, however, we observed cardiac CD36 mRNA and protein expression to ensure the efficacy of lentivirus-mediated RNAi. Hence, we have sound reasons to believe that the beneficial results observed in this study are mainly attributed to the downregulation of cardiac CD36.

Although obesity is commonly considered a risk factor in the development of cardiovascular disease, a recent study suggested that it may also be associated with increased tolerance to ischaemia in isolated heart - the so-called “obesity paradox”[31]. This finding is in stark contrast to previous findings in obese model on a similar diet. It has been repeated proved by clinical and basic studies that obesity would adversely affect cardiac remodeling and heart failure, which is closely related to myocardial lipid metabolic disorder. We do not suspect that FA would play a cardioprotection role under certain conditions because it provides the majority of ATP required for heart contraction. Nevertheless, increased FA metabolism associated with obesity is the primary cause of the deleterious effects on cardiac remodeling and systolic function in this study.

In another investigation by Zhong Q and colleagues, high density lipoprotein increased oxidized low density lipoprotein (LDL) uptake of inflammatory adipocytes via upregulation of PPARγ/CD36 pathway, which may be a new mechanism of anti-atherosclerosis [46]. This study aimed at increasing oxidized LDL uptake of adipocytes, which in turn would reduce serum level of LDL, thereby inhibiting the process of atherosclerosis. The research strategy of this study is different from ours. This study utilized adipocytes to ingest excessive LDL, whereas our intervention strategy aimed at limiting CD36-mediated excessive myocardial FA intake under the condition of FA oversupply, thus protecting the heart from remodeling and dysfunction. Therefore, it is not surprising to find different results when regulate the activity or expression of CD36 under different pathophysiological conditions with different methods in different tissues.

Nevertheless, approximately half of the studies showed no significant difference in LV systolic function between obese and lean patients [4, 47, 48], whereas the remainder showed significantly lower mean LVEF or LVFS values in obese than in lean subjects [3, 5, 6]. We speculate that the strength of association between severity of obesity and cardiac damage is stronger in animal models, in which myocardial metabolism can be operate in a more extreme (though unphysiological) pattern.

### Limitations

There are limitations in our study for identifying the exact mechanisms underlying the reduction of serum levels of BUN, creatinine and triglyceride with cardiospecific CD36 inhibition. Further studies aimed at the mechanism underlying this link are necessary to substantiate this conclusion. Besides, a comparative study with cardiospecific CD36 overexpression is required to elucidate the specific effect of CD36 in obesity cardiomyopathy.

## Conclusions

Collectively, the present study demonstrates the protective role of cardiospecific CD36 inhibition against obesity cardiomyopathy, as modeled by HFD fed mice. The morphological and functional improvements of the hearts may be mediated by the alleviation of lipid accumulation and reversal of ROS generation. These observations imply that the treatment strategies for cardiac dysfunction should have a metabolic character. Inhibition of cardiac CD36 may serve as a potential approach for treatment of obesity cardiomyopathy.

## Abbreviations

ALP: Alkaline phosphatase
ALT: Alanine aminotransferase
AST: Aspartate transaminase
BUN: Blood urea nitrogen
CND: Control-normal-diet
DCFH-DA: 2’,7’-dichlorofluorescein diacetate
DHE: Dihydroethidium
FA: Fatty acid
FSG: Fasting serum glucose
GFP: Green fluorescent protein
HFD: High-fat-diet
LDL: Low density lipoprotein
LV: Left ventricular
LVEDd: Left ventricular end-diastolic diameter
LVEF: Left ventricular ejection fraction
LVESd: Left ventricular end-systolic diameter
LVFS: Left ventricular fractional shortening
LVPWd: Left ventricular posterior wall thickness at end-diastole
LVPWs: Left ventricular posterior wall thickness at end-systole
ROS: Reactive oxygen species
shRNAs: Short hairpin RNAs
TC: Total cholesterol
